# Evaluation of Fecal Calprotectin, D-Lactic Acid and Bedside Gastrointestinal Ultrasound Image Data for the Prediction of Acute Gastrointestinal Injury in Sepsis Patients

**DOI:** 10.3389/fmedt.2021.733940

**Published:** 2021-11-01

**Authors:** Junlu Li, Yanbo Ren, Chang Gao, Kaili Zhang, Fuwen Zheng, Jian Kang

**Affiliations:** Emergency Department, First Affiliated Hospital of Dalian Medical University, Dalian, China

**Keywords:** fecal calprotectin, sepsis, acute gastrointestinal injury, D-Lactic acid, bedside gastrointestinal ultrasound

## Abstract

**Objective:** To investigate the early warning and prognostic evaluation of fecal calprotectin (FC), D-lactic acid, and bedside gastrointestinal ultrasound (B-GIUS) data for acute gastrointestinal injury (AGI) in sepsis patients.

**Main Method:** Sepsis patients were grouped based on the presence or absence of AGI into AGI and non-AGI groups. Healthy volunteers of the same period were selected as the control group. FC, B-GIUS data, D-lactic acid, etc. were collected on the 1st, 3rd and 7th days of admission. Twenty-eight-day mortality was recorded.

**Main Results:** FC, D-lactic acid levels, gastric antrum cross-sectional area, and small intestine wall thickness were significantly increased in group A and B (*P* < 0.05); furthermore, inner-to-outer diameter ratio and cross-sectional area of small intestine were lower than those in the control group (*P* < 0.05). FC, D-lactic acid, gastric antrum cross-sectional area and small intestine wall thickness in AGI group were higher than those in non-AGI group (*P* < 0.05). Inner-to-outer diameter ratio and cross-sectional area of small intestine in AGI group were smaller than those in non-AGI group (*P* < 0.05). There was no difference in the thickness, inner-to-outer diameter ratio nor the cross-sectional area ratio of colon between AGI and non-AGI groups (*P* > 0.05). AUC for D-lactic acid was 0.881, which was higher than FC's (0.74). When the D-lactic acid cutoff value was 22.16 μmol/L, the sensitivity was 77.9% and the specificity was 92% for the prediction of AGI in sepsis. AUC for the cross-sectional area of the gastrointestinal antrum was 0.657, which was higher than the small intestine thickness's (0.629). When the gastric antrum cross-sectional area was larger than 4.20 cm^2^, the sensitivity was 64% and the specificity was 65.3%.

**Conclusion:** D-Lactic acid and FC were early diagnostic indicators for sepsis with AGI, and D-lactic acid was the superior indicator. The gastric antrum cross-sectional area and the small intestine wall thickness had an early warning effect, and the prediction of the gastric antrum cross-sectional area was superior to that of the latter. Because it is non-invasive and convenient, B-GIUS can help in the diagnosis of sepsis with AGI.

## Introduction

Sepsis is a life-threatening organ dysfunction caused by host response imbalance (1). The intestine is the initiating and target organ of sepsis (2). The evaluation of acute gastrointestinal injury and its severity in a timely, accurate and objective manner plays an important role in sepsis therapy. At present, the diagnosis of acute gastrointestinal injury (AGI) is mainly based on clinical symptoms, cumbersome and subjective and lacks reliable and accurate standards.

Calprotectin, an inflammatory marker derived and released from neutrophils and macrophages after activation, is widely distributed in various cells, tissues and body fluids (3, 4). Because the stool directly contacts the intestinal mucosa, the level of fecal calprotectin (FC) can accurately reflect inflammation and exudation (5).

D-Lactic acid is a metabolite of intestinal bacteria. When the body is severely traumatized, shocked, etc., the intestinal mucosal barrier is damaged, and the permeability increases. D-Lactic acid enters the blood circulation though the damaged intestinal mucosa; therefore, the blood D-lactic acid level increases. Studies have found that D-lactic acid gradually increases with the development of damage to intestinal function and diseases (6), meaning D-lactic acid levels can be used as an indicator for clinical testing of the permeability of the intestinal barrier and bacterial translocation (7). Since Bateman used gastrointestinal ultrasound to measure gastric emptying in 1982, gastrointestinal ultrasound has been used in the clinic (8), and this technology has been further developed in recent years.

Therefore, the aim of this experiment was to monitor the levels of FC and D-lactic acid in sepsis patients and explore the role of FC and D-lactic acid in the early warning and prognostic evaluation of sepsis with AGI. At the same time, a new method was provided that offers safe, real-time and dynamic characteristics of bedside gastrointestinal ultrasound (GIUS) for evaluating the gastrointestinal function of sepsis.

## Materials and Methods

### Subjects

A prospective research method was used in this study. Sepsis patients who were admitted to the EICU of the First Affiliated Hospital of Dalian Medical University from January 2019 to December 2019 and met the diagnostic criteria of “Sepsis 3.0” (9) (*n* = 54) were included in the test group; 33 were males, and 21 were females, with an average age of 66.20 ± 13.63 years. Healthy volunteers from the same period were selected as the control group (*n* = 15), including eight males and seven females, with an average age of 58.27 ± 13.46 years.

Exclusion criteria: ① patients aged <18 years; ② pregnant and lactating women; ③ patients with primary acute and chronic gastrointestinal diseases; ④ patients receiving radiotherapy and chemotherapy; ⑤ patients receiving immunosuppressive therapy within 3 months; ⑥ HIV patients; ⑦ patients with severe liver insufficiency; ⑧ patients undergoing colonoscopy or receiving antibiotic treatment 1 week before admission; and ⑨ patients with incomplete clinical data. This study was approved by the Ethics Committee of the First Affiliated Hospital of Dalian Medical University (PJ-KS-KY-2019-60, PJ-XJS-2018-49), and informed consent was obtained from the patients or family members.

### Research Method

Data for all the selected sepsis patients were recorded, including general basic information, and patients were divided into the AGI group (group A) (*n* = 29) and the non-AGI group (group B) (*n* = 25) based on AGI grade according to the definition of “2012 ESICM Recommendations: Terminology, Definition and Management of Gastrointestinal Function in Critically Ill Patients” (10). Venous blood was drawn on the 1st, 3rd, and 7th days after admission; a routine blood test was performed; and levels of D-lactic acid, IL-6, procalcitonin, etc. were determined. SOFA and APACHE II scores were calculated; enrolled patients' symptoms and physical signs, such as vomiting, hiccups, abdominal pain, abdominal distension, diarrhea, constipation, bowel sounds, and gastrointestinal bleeding, were monitored; feces were collected for fecal calprotectin and stool routine tests; and bedside GIUS was performed on an empty stomach before dinner to detect the cross-sectional area of the gastric antrum (directly measured by a GE machine) and the thickness of the small intestine and outer and inner colon. In the control group, the indexes of the venous blood, feces, and GIUS data were also collected while performing routine physical examination. D-Lactic acid levels were determined using a Human D-Lactic acid (D-LA) ELISA Kit from Shanghai Enzyme-linked Biotechnology. The FC test was performed using the Calprotectin Detection Kit (Chromatographic Assay of Colloidal Gold) from Mokobio Life Science Corporation Beijing, and GIUS was performed by the LOGIQe ultrasound machine from GE Healthcare.

### Statistical Method

This experiment used SPSS 23.0 statistical analysis software for data processing and analysis. The Kolmogorov-Smirnov test method was used to test the independent and dependent variables. The measurement data that met the normal distribution are expressed as the mean ± standard deviation ($\bar{x}$ ± s), and an independent-sample T test or analysis of variance was used to compare differences between groups. Variables that did not meet the normal distribution are expressed as the median (25% quantile and 75% quantile). The Mann-Whitney U nonparametric test was used to compare differences between groups. The Kruskal-Wallis test was used to compare the differences between groups. The statistical test was a two-sided test. α = 0.05 was used as the test standard, and *P* < 0.05 was considered statistically significant. The ROC curve was drawn, the area under the curve (AUC) was calculated, the early diagnostic value of FC and D-lactic acid for sepsis with AGI was evaluated, and the best cutoff point was determined.

## Results

### General Information

A total of 54 sepsis patients were enrolled. There was no significant difference in age or sex composition among the sepsis with AGI group (group A), the non-AGI group (Group B) and the control group (Group C) (*P* > 0.05) (Table 1).

**Table 1:** General information.

**Indexes**	**Group A (*n* = 29)**	**Group B (*n* = 25)**	**Group C (*n* = 15)**	***P value***
Sex (male/female)	17/12	16/9	8/7	*P* > 0.05
Age	66.35 ± 14.13	66.04 ± 13.31	58.27 ± 13.46	*P* > 0.05

### Comparison of D-Lactic Acid and Fecal Calprotectin Levels in the AGI, Non-AGI and Control Groups

**Figure 1:**
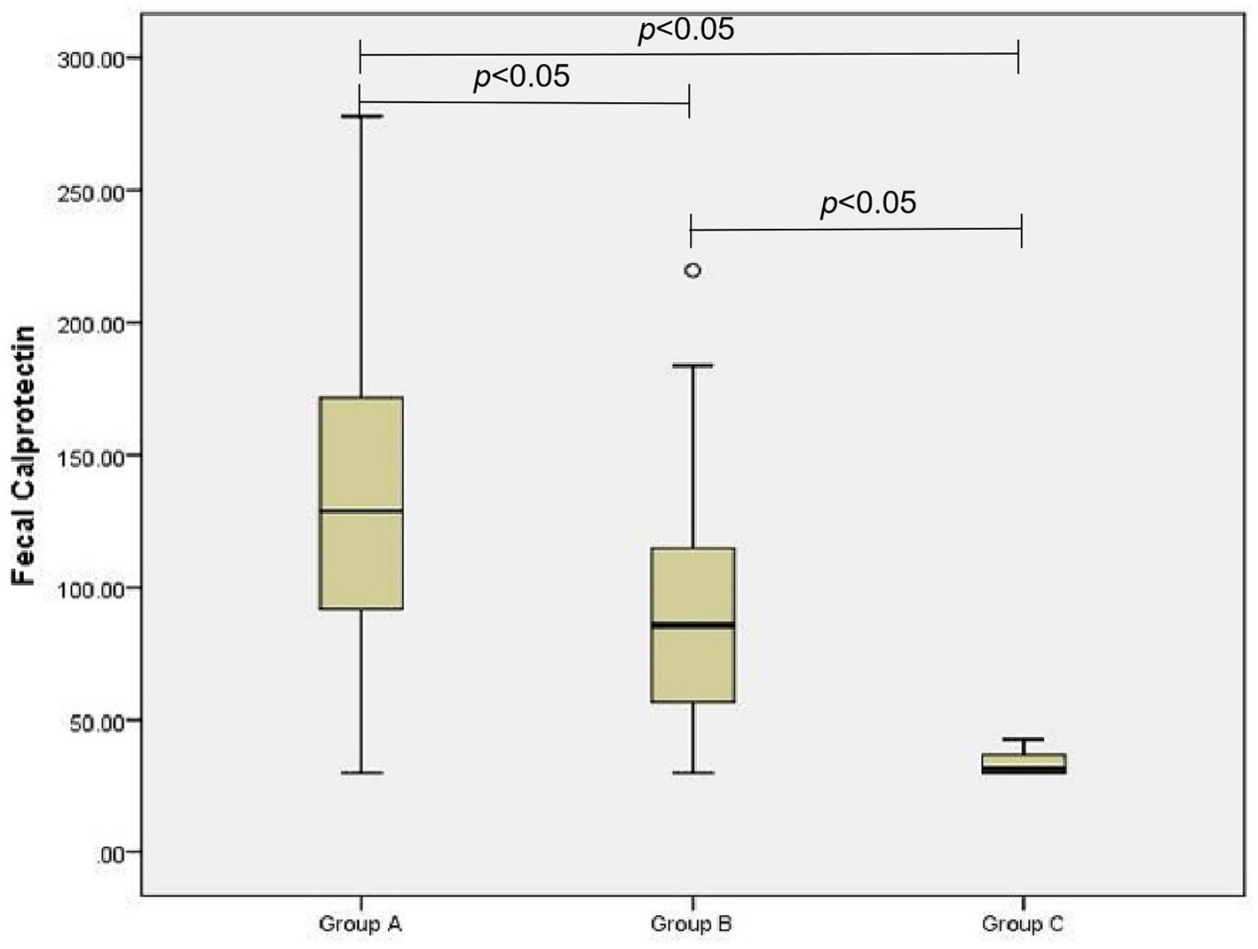
Comparison of fecal calprotectin levels in group A, B, and C.

**Figure 2:**
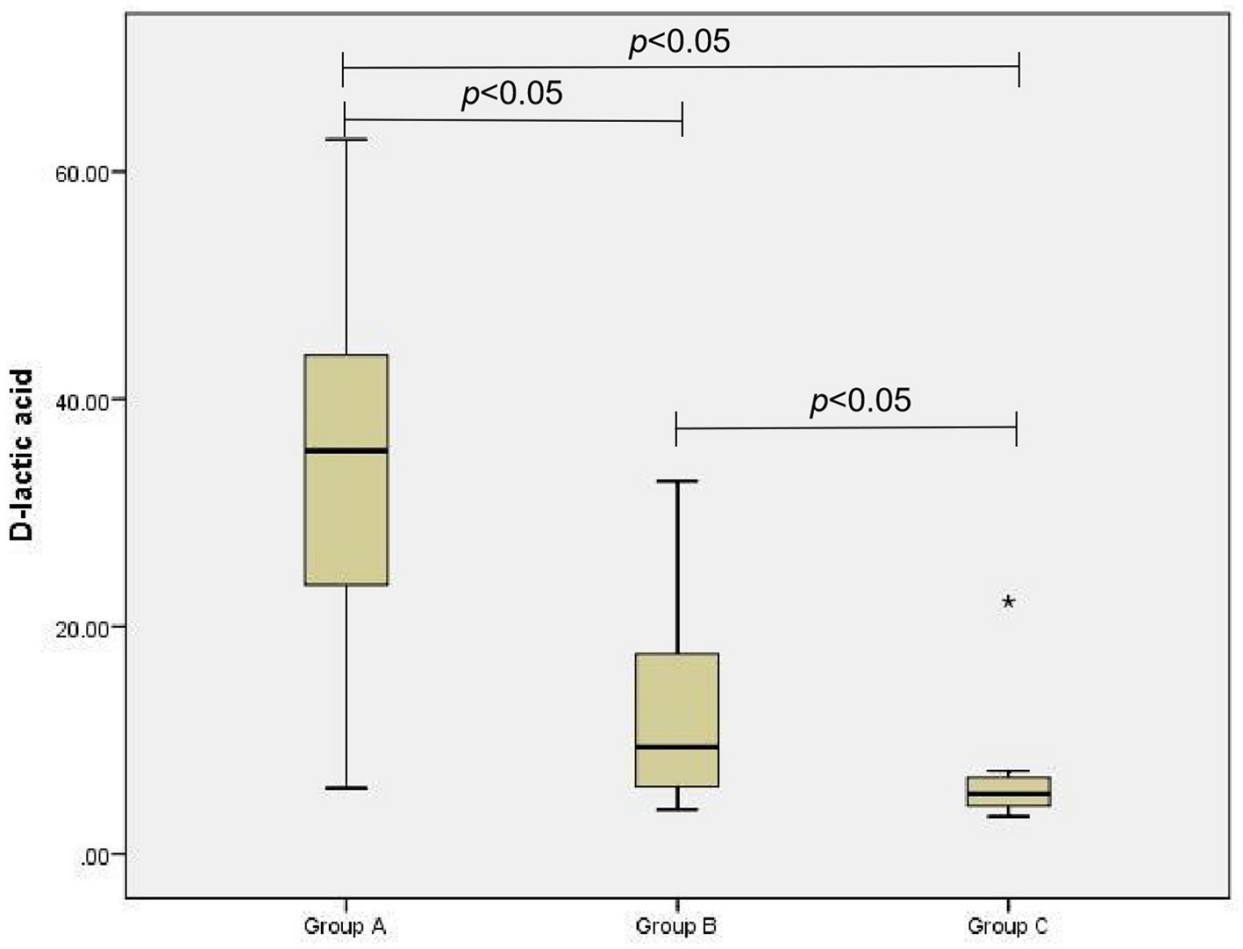
Comparison of D-lactic acid levels in group A, B, and C.

The D-lactic acid and FC levels of the AGI and non-AGI groups were significantly higher than those in the control group (both *P* < 0.05); the D-lactic acid and FC levels of the AGI-group were significantly higher than those in the non-AGI group (both *P* < 0.05) (Figures 1, 2).

### Comparison of the Gastric Antrum Cross-Sectional Area, Wall Thickness, Inner-to-Outer Diameter Ratio, and Cross-Sectional Area of the Small Bowel and Colon of the Three Groups

According to the Kruskal-Wallis test and pairwise comparison, the cross-sectional area of the gastric antrum and the wall thickness of the small intestine and colon in group A were larger than those in group B, and those of both group A and group B were larger than those in group C (*P* < 0.05 after adjustment). The inner-to-outer diameter ratio and the cross-sectional area of the small intestine and colon of groups A and B were lower than those in group C (*P* < 0.05 after adjustment). The inner-to-outer diameter ratio and cross-sectional area of the small intestine in group A were lower than those in group B (*P* < 0.05 after adjustment). However, the wall thickness, inner-to-outer diameter ratio and cross-sectional area of the colon did not differ between groups A and B (*P* > 0.05 after adjustment) (Table 2).

**Table 2:** Comparison of the gastrointestinal ultrasound data of the three groups.

**Indexes**	**Group A**	**Group B**	**Group C**	***P1***	***P2***	***P3***
Gastric antrum cross-sectional area (cm^2^)	4.87 (3.67, 6.73)	3.82 (3.25, 4.86)	2.44 ± 0.79	*P* = 0.016	*P* < 0.001	*P* < 0.001
Small intestine wall thickness (cm)	0.35 (0.22, 0.49)	0.26 (0.21, 0.33)	0.22 (0.19, 0.26)	*P* = 0.027	*P* < 0.001	*P* = 0.002
Ratio of inner-to-outer diameter of small intestine (%)	68.07 (57.59, 77.31)	73.53 (67.28, 80.44)	80.83 ± 4.52	*P* = 0.027	*P* < 0.001	*P* < 0.001
Ratio of cross-sectional area of small intestine (%)	48.53 ± 17.66	54.98 ± 13.95	65.6 (63.4, 72.4)	*P* = 0.018	*P* < 0.001	*P* < 0.001
Colon wall thickness (cm)	0.42 (0.31, 0.56)	0.38 (0.28, 0.51)	0.29 ± 0.5	*P* > 0.05	*P* < 0.001	*P* < 0.001
Ratio of inner-to-outer diameter of colon (%)	74.92 (64.78, 79.89)	76.31 (68.79, 82.07)	82.49 (74.75, 85.64)	*P* > 0.05	*P* < 0.001	*P* = 0.003
Ratio of cross-sectional area of colon (%)	61.50 (47.80, 68.90)	60.40 (51.80, 69.20)	67.16 ± 7.29	*P* > 0.05	*P* = 0.008	*P* = 0.01

*P1, P value of group A and B; P2, P value of group A and C; P3, P value of group B and C*.

### Correlation Results Between Fecal Calprotectin, D-Lactic Acid and SOFA Score, APACHE II Score, and IL-6 in the AGI and Non-AGI Groups

In all selected sepsis patients, regardless of AGI status, the FC value was positively correlated with D-lactic acid, IL-6, and SOFA and APACHE II scores (*P* < 0.05); D-lactic acid was positively correlated with IL-6, SOFA and APACHE II scores in the AGI group (*P* < 0.05) (Tables 3, 4).

**Table 3:** Correlation results of FC with IL-6, SOFA score and APACHE II score.

**Indexes**	**Group A**		**Group B**	
	**r**	***P* value**	**r**	***P* value**
D-Lactic acid	0.432^a^	<0.001	0.452^b^	<0.001
IL-6	0.373^b^	<0.001	0.27^b^	<0.001
SOFA score	0.422^b^	<0.001	0.593^b^	<0.001
APACHE II score	0.402^a^	<0.001	0.468^b^	<0.001

a *Pearson's correlation coefficients*;

b *Spearman's correlation coefficients; SOFA, Sequential Organ Failure Assessment; APACHE II, Acute Physiology and Chronic*.

**Table 4:** Correlation results of D-lactic acid with IL-6, SOFA score and APACHE II score.

**Indexes**	**Group A**		**Group B**	
	**r**	***P* value**	**r**	***P* value**
IL-6	0.368^b^	<0.001	0.025^b^	0.834
SOFA score	0.301^b^	0.005	0.271^b^	0.019
APACHE II score	0.298^a^	0.005	0.2^b^	0.085

a *Pearson's correlation coefficients*;

b *Spearman's correlation coefficients; SOFA, Sequential Organ Failure Assessment; APACHE II, Acute Physiology and Chronic Health Evaluation*.

### The Predictive Value of D-Lactic Acid, Fecal Calprotectin, IL-6, Gastric Antrum Cross-Sectional Area, and Small Intestinal Wall Thickness in Sepsis With Acute Gastrointestinal Injury

**Figure 3:**
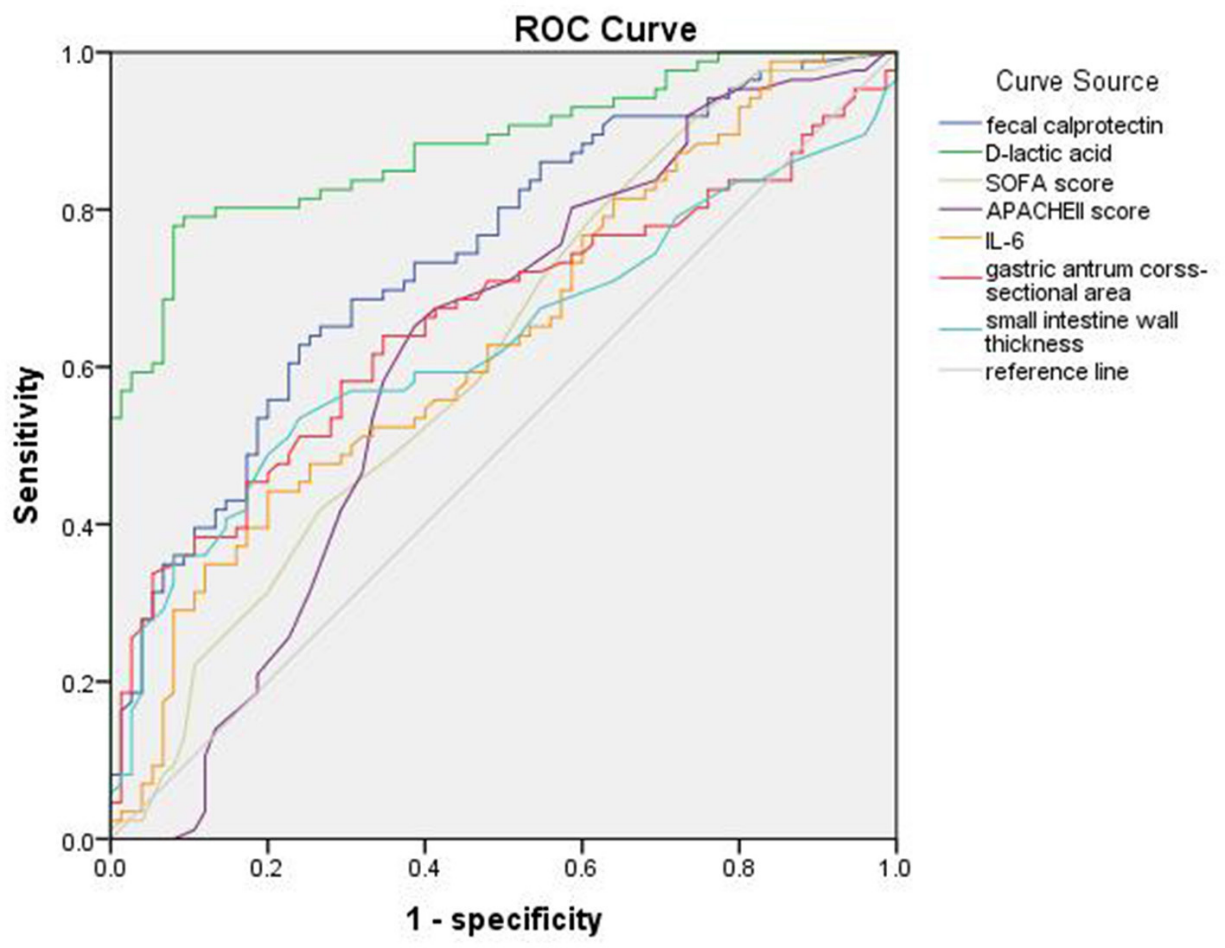
ROC curve of fecal calprotectin, D-lactic acid, SOFA score, APACHEII score, IL-6, gastric antrum cross-sectional area and small intestine wall thickness of sepsis with AGI.

The ROC curves of D-lactic acid, FC, IL-6, gastric antrum cross-sectional area, small intestine wall thickness, etc. for predicting sepsis with AGI are shown in Figure 3. The AUC of D-lactic acid was 0.881, which was higher than that of FC (0.74, *P* < 0.05), IL-6 (0.635, *P* < 0.05) and the cross-sectional area of the gastric antrum (0.657, *P* < 0.05). When the cutoff value of D-lactic acid was 22.16 μmol/L, the sensitivity of D-lactic acid in predicting sepsis with AGI was 77.9%, and the specificity was 92%. The AUC of the gastric antrum cross-sectional area was 0.657, which was higher than that of the small intestine wall thickness (0.629, *P* < 0.05). When the gastric antrum cross-sectional area was greater than 4.20 cm^2^, the sensitivity was 64%, and the specificity was 65.3% for the prediction of sepsis with AGI (Table 5).

**Table 5:** AUC of the indicators of sepsis with AGI.

**Indexes**	**AUC**	**Standard error^a^**	**Coef^b^**	**95% CI**		**Cutoff value**	**Sensitivity**	**Specificity**
				**Lower limit**	**Upper limit**			
Fecal calprotectin	0.74	0.039	0	0.665	0.816	115.25	62.80%	76%
D-Lactic acid	0.881	0.026	0	0.83	0.933	22.16	77.90%	92%
IL-6	0.635	0.044	0.01	0.549	0.72	140.02	44.20%	80%
Gastric antrum cross-sectional area	0.657	0.046	0.014	0.573	0.742	4.2	64%	65.30%
Small intestine wall thickness	0.629	0.044	0.003	0.542	0.716	0.34	54%	76%
SOFA score	0.618	0.043	0.001	0.531	0.705	6	79.10%	38.70%
APACHE II score	0.613	0.044	0.005	0.523	0.703	19	65.10%	61.30%

a *Assumed by non-parameters*.

b *Null hypothesis: true area = 0.5*.

## Discussion

The gastrointestinal tract is the location of the initiation of multiple organ failure and one of the first systems affected. The high fatality rate of sepsis with acute gastrointestinal injury has attracted increasing attention. At present, there is no reliable laboratory indicator for diagnosing acute gastrointestinal injury in sepsis patients. In this study, we used a prospective experiment to investigate the effects of fecal calprotectin, D-lactic acid, and bedside GIUS data on the early warning and evaluation of sepsis with AGI. The results showed that both D-lactic acid and FC were early predictive indicators for sepsis with AGI, and D-lactic acid was a much more valuable indicator. The changes in the gastric antrum cross-sectional area and the thickness of the small intestine wall were early warning indicators for sepsis with AGI, and the former was much more valuable.

Fecal calprotectin is a calcium-containing protein released after the activation of neutrophils and macrophages; is widely distributed in human cells, tissues and bodily fluids; and can be used as a marker of acute inflammatory cell activation (11). The concentrations of FC are different in different locations (12), as are the tissue and cell expression and specificity. Because stool is in contact with the intestinal mucosa and FC survives stably in stool, FC levels can more accurately reflect the inflammation and exudation of the intestinal mucosa than calprotectin in blood. At present, FC has been used worldwide to initially screen for IBD, IBS and colorectal cancer (13). Sepsis leads to intestinal ischemia and hypoxia, inflammatory reactions, intestinal barrier function impairment, intestinal flora imbalance, bacterial translocation and endotoxemia (14). The results of this study showed that FC levels in the sepsis with AGI group were significantly higher than those in the non-AGI group, indicating that the intestinal barrier function was impaired and that the inflammatory reaction more seriously occurred when sepsis was combined with acute gastrointestinal injury.

D-Lactic acid is a metabolite of fermentation by bacteria inherent in the gastrointestinal tract. Other tissues can neither produce nor metabolize this metabolite, so D-lactic acid in blood is derived from the intestine (15). When the body experiences sepsis, D-lactic acid enters the blood circulation through the damaged intestinal mucosa. The results of this experiment showed that the plasma D-lactic acid levels in the sepsis with AGI group were significantly higher than those in the other two groups, which was consistent with previous research results. As the severity level of intestinal injury increases, plasma D-lactic acid increases significantly (6, 16).

According to the results of this experiment, the AUC of FC was 0.74 (95% CI 0.665, 0.816). When the cutoff value of FC was 115.25 μg/g, the sensitivity was 62.8%, and the specificity was 76% for the prediction of AGI in sepsis. The AUC of D-lactic acid was 0.881 (95% CI 0.83–0.933). When the cutoff value of D-lactic acid was 22.16 μmol/L, the sensitivity was 77.9%, and the specificity was 92% for the prediction of AGI in sepsis. All of these results indicated that FC and D-lactic acid were both valuable in predicting sepsis with AGI, but D-lactic acid was much more valuable.

Since 1985, Bolondi et al. used only changes in the gastric antrum area under ultrasound to determine gastric emptying in patients (17), and the value of the clinical application of GIUS has gradually been discovered. At present, a single transverse section of the gastric antrum with bedside ultrasonography is used to measure the antral contraction amplitude (ACA), antral contraction frequency (ACF), antral motility index (MI), and gastric emptying time (GET) as objective indicators for evaluating gastric emptying function to guide clinical practice in severe patients (18). However, since the time for the examination of critical patients in the emergency department should not be too long in actual clinical operations, this experiment was adopted to observe the early changes in the cross-sectional area of the gastric antrum of the patients. To avoid measurement errors caused by the irregularity of the gastric antrum, we used a GE ultrasound machine to directly measure the cross-sectional area of the gastric antrum instead of the diameter on the coronal plane and the sagittal plane. The results of the study showed that the cross-sectional area of the gastric antrum in the AGI group increased significantly. When the area was larger than 4.20 cm^2^, it was an early warning indicator for sepsis with acute gastrointestinal injury.

At present, a diameter of the small intestine >3 cm and a diameter of the colon >6 cm (cecum > 9 cm) measured by abdominal CT scan are indicative of intestinal dilatation (19), but there is still no authoritative standard description for the quantitative standard of intestinal ultrasound. Studies of IBD have shown that, in general, intestinal wall thickness >0.3 cm can be considered intestinal wall thickening, and the standard of intestinal wall thickness >0.4 cm is more specific for the diagnosis of IBD (20). In this study, it was found that the median value of small intestinal wall thickness of the sepsis with AGI group was 0.35 (0.22, 0.49) cm, and the colon wall thickness was 0.42 (0.31, 0.56) cm, which were both >0.3 cm, which indicated that the walls of the small intestine and colon in sepsis patients with AGI were thickened. We found that due to the different intestinal contents of some patients, the intestinal tube had various shapes, such as oval, narrow, and flat. The intestinal tube is not completely cylindrical. To reduce the error in obtaining the diameter of the intestinal lumen, this experiment innovatively added the cross-sectional area of the inner-outer intestines and expressed the overall morphological changes in the intestinal tube by the ratio of the coplanar small/colon intestinal cross-sectional area. We found that the inner-to-outer cross-sectional area ratio in sepsis patients was significantly lower than that in healthy people. Moreover, this ratio in sepsis patients with AGI was lower than that in sepsis patients without AGI, which once again proved that there were morphological changes in the intestine in the early stage of sepsis. According to the intestinal mucosal barrier damage in the early stage of sepsis, the early increase in IL-6 (as a proinflammatory factor), FC and D-lactic acid in this experiment can indicate that there may be inflammatory edema of the intestinal wall in the early stage of sepsis. In summary, we found that patients with sepsis with AGI had early morphological changes, such as an increase in the cross-sectional area of the gastric antrum, a thickening of the small intestine wall, and a decrease in the small intestine inner-to-outer diameter ratio and cross-sectional area ratio. We additionally found that the AUC of the intestinal inner-to-outer diameter ratio and the cross-sectional area ratio were <0.5, which meant that both diagnostic values were low and that the clinical diagnostic significance of AGI was relatively limited. There is a need for larger-scale research in the future.

Above all, we concluded that D-lactic acid, FC, gastric antrum cross-sectional area, and small intestinal wall thickness were early warning indicators for sepsis with acute gastrointestinal injury. Bedside GIUS, due to its non-invasiveness and convenience, can help in the clinical diagnosis of sepsis with acute gastrointestinal injury.

Up to now the evaluation of AGI is relatively cumbersome and there is no unified index. The advantages of this experiment are the following (21–25): fecal calprotectin as an index was used innovatively for clinical diagnosis of sepsis with AGI in this experiment. Because feces directly contacts the intestine, it is more direct, fast, convenient and non-invasive than blood samples. The bedside ultrasound examination can save time for sonographers to arrive at the scene and get the condition of the clinician, ensure the pertinence of the examination, and can be carried out at the same time as the rescue, which effectively increases the efficiency of the evaluation of the condition, greatly saves the rescue time, evaluates the patient's condition and provides a reliable basis for diagnosis and identification. It avoids the deterioration, respiratory or cardiac arrest and even death caused by the inspection and transportation of the patient. It is especially suitable for trauma and other patients with transportation contraindications or unstable circulation. Bedside ultrasound has the advantages of mobile, non-invasive, simple and practical, fast and safe, dynamic comparison, can be used for children, pregnant women. It is suitable for emergency and intensive care departments. The indicators in this experiment have a relatively effective, rapid and non-invasive early warning effect on acute gastrointestinal injury.

This experiment has certain limitations. First, this was a single-center study. Second, the number of patients included in the experiment was small, which may cause bias in the results. Third, this study found the following three states through ultrasound exploration of the intestinal morphology of sepsis: ① the intestine was relatively expanded and the intestinal wall was thin; ② the intestine was relatively narrow and the intestinal wall was thickened; and the intestine was dilated and ③ the intestinal wall was thickened. In this study, we used the methods required by the “Technical specification for clinical application of critical ultrasonography-Gastro-intestine” issued by the Chinese Critical Ultrasound Study Group and Critical Hemodynamic Therapy Collaboration Group (26). Since there was no standard intestinal ultrasound quantification value, the three states were expressed by the inner-to-outer diameter ratio and the ratio of the cross-sectional area of the intestine. However, in special circumstances, such as in cases of severe intestinal gas, we placed the probe on the bilateral mid-axillary section instead of the scanning section of the flank colon, placed the probe on the posterior axillary line and then slid up and down for scanning, or we placed the probe under the cut surface of the liver and spleen by finding the liver and spleen area of the colon as a fixed landmark area and then slid down along the cut surface of the intestine to increase the success of intestinal exploration. However, due to the small sample size in this experiment, the intestinal morphology classification cannot be made on the basis of the type or the severity level of the disease, and because most critical patients suffer from multiple diseases at the same time, the variables cannot be tightly controlled. Therefore, it is necessary to continuously expand the sample size and develop multicenter research to verify the intestinal morphology and functional status of different diseases, as well as the technical path of ultrasound probes to explore the intestinal tract.

## Data Availability Statement

The raw data supporting the conclusions of this article will be made available by the authors, without undue reservation.

## Ethics Statement

The studies involving human participants were reviewed and approved by Ethics Committee of the First Affiliated Hospital of Dalian Medical University. The patients/participants provided their written informed consent to participate in this study.

## Author Contributions

JK, YR, and JL contributed to the conception of the work. JL, YR, and FZ screened patients and assessed eligibility. JL, CG, and KZ conducted patient interviews and administered surveys. JL and YR performed physical examinations. JL created all figures and performed the data analysis. JL and YR drafted the manuscript and critically revised the paper. All authors contributed to the article and approved the submitted version.

## Conflict of Interest

The authors declare that the research was conducted in the absence of any commercial or financial relationships that could be construed as a potential conflict of interest.

## Publisher's Note

All claims expressed in this article are solely those of the authors and do not necessarily represent those of their affiliated organizations, or those of the publisher, the editors and the reviewers. Any product that may be evaluated in this article, or claim that may be made by its manufacturer, is not guaranteed or endorsed by the publisher.
